# Heritability of physical activity traits in Brazilian families: the Baependi Heart Study

**DOI:** 10.1186/1471-2350-12-155

**Published:** 2011-11-29

**Authors:** Andréa RVR Horimoto, Suely R Giolo, Camila M Oliveira, Rafael O Alvim, Júlia P Soler, Mariza de Andrade, José E Krieger, Alexandre C Pereira

**Affiliations:** 1 Laboratory of Genetics and Molecular Cardiology, Heart Institute, Medical School of University of São Paulo, Av. Dr. Enéas de Carvalho Aguiar, 44, São Paulo, SP, 05403-000, Brazil; 2 Department of Statistics, Polytechnic Center, Federal University of Paraná, Av. Cel. Francisco H. Santos, 100, Curitiba, PR, 81531-990, Brazil; 3 Department of Statistics, Mathematics and Statistics Institute, University of São Paulo, R. do Matão, 1010, São Paulo, SP, 05508-090, Brazil; 4 Department of Health Sciences Research, Mayo Clinic, 200 First Street S.W., Rochester, MN, 55905, USA

## Abstract

**Background:**

It is commonly recognized that physical activity has familial aggregation; however, the genetic influences on physical activity phenotypes are not well characterized. This study aimed to (1) estimate the heritability of physical activity traits in Brazilian families; and (2) investigate whether genetic and environmental variance components contribute differently to the expression of these phenotypes in males and females.

**Methods:**

The sample that constitutes the Baependi Heart Study is comprised of 1,693 individuals in 95 Brazilian families. The phenotypes were self-reported in a questionnaire based on the WHO-MONICA instrument. Variance component approaches, implemented in the SOLAR (Sequential Oligogenic Linkage Analysis Routines) computer package, were applied to estimate the heritability and to evaluate the heterogeneity of variance components by gender on the studied phenotypes.

**Results:**

The heritability estimates were intermediate (35%) for weekly physical activity among non-sedentary subjects (weekly PA_NS), and low (9-14%) for sedentarism, weekly physical activity (weekly PA), and level of daily physical activity (daily PA). Significant evidence for heterogeneity in variance components by gender was observed for the sedentarism and weekly PA phenotypes. No significant gender differences in genetic or environmental variance components were observed for the weekly PA_NS trait. The daily PA phenotype was predominantly influenced by environmental factors, with larger effects in males than in females.

**Conclusions:**

Heritability estimates for physical activity phenotypes in this sample of the Brazilian population were significant in both males and females, and varied from low to intermediate magnitude. Significant evidence for heterogeneity in variance components by gender was observed. These data add to the knowledge of the physical activity traits in the Brazilian study population, and are concordant with the notion of significant biological determination in active behavior.

## Background

Physical inactivity has been identified as the fourth leading risk factor for global mortality (6% of deaths worldwide), preceded only by high blood pressure (13%), tobacco use (9%) and high blood glucose (6%). Inactivity is associated with several diseases, such as cardiovascular disease, type 2 diabetes, obesity, hypertension, colon and breast cancer, anxiety and depression [1].

Numerous epidemiologic studies have emphasized the importance of regular physical activity in the prevention and treatment of several common chronic diseases. Despite the benefits of physical activity on health, levels of inactivity are high in virtually all developed and developing countries. At least 60% of the world population presents a sedentary behavior, mainly because of insufficient participation in physical activity during leisure time and a decrease in occupational and domestic activities [2]. In Brazil, from 10.7% to 22.1% of the adult population was classified as completely inactive, with indexes higher in males (15.0%) than in females (13.6%); the maximum frequency of inactivity was in the age group over 65 years old (35.8% in males and 37.7% in females) [3].

Participation in physical activity is a behavioral trait determined by environmental and genetic factors that may reflect, in part, the predisposition to adopt and maintain a physically active lifestyle. Among adults, physical activity includes leisure time physical activity, transportation (e.g. walking or cycling), occupational activity (i.e. work), household chores, and participation in games, sports or planned exercise, all in the context of daily, family, and community activities. There are specific recommendations of physical activity for healthy adults in the age ranges of 18-65 years and 65 years and above to promote and maintain health which take into account the quantity (in minutes) and the intensity of the activity [2,4].

Familial aggregation of distinct physical activity dimensions, such as total or weekly physical activity, leisure time activity, and level of daily physical activity [5,6] has been suggested mainly from twin and family studies. Diversity in sample size, age, gender, phenotype definition, and statistical procedures among the different studies probably explains why heritability estimates have ranged from 30% to 83% in twin studies and from 10% to 30% in studies with nuclear families [7]. Nonetheless, heritability accounts for a substantial portion of the variation of physical activity phenotypes that have been reported at the population level.

In this study, we estimated the genetic influences on sedentarism, weekly physical activity (exclusively related to participation in sports activities), weekly physical activity among non-sedentary subjects, and level of daily physical activity (related to work and free-time activities, excluding sports) in the families that participated in the Baependi Heart Study. We also investigated whether genetic and environmental components contribute differently to the expression of these phenotypes in males and females.

## Methods

### The Baependi Heart Study

The Baependi Heart Study [8] is a genetic epidemiological study of cardiovascular disease risk factors, with a longitudinal design. Baseline enrollment occurred between December 2005 and January 2006 when 1,857 individuals distributed in 95 families resident in the municipality of Baependi, a city located in the Southeast of Brazil were selected to participate in the study. Probands were identified, in several stages, from the community at large. Eleven census districts (from a total of twelve) were selected for the study and the residential addresses within each district were randomly selected (first by randomly selecting a street and then a household). Only subjects age 18 and older, living in the selected household, were eligible to participate in the study.

When a proband was enrolled, all his/her first-degree (parents, siblings, and offspring), second-degree (half-siblings, grandparents/grandchildren, uncles/aunts, nephews/nieces, and double cousins), and third-degree (first-cousins, great-uncles/great-aunts, and great-nephews/great-nieces) relatives and the relatives of their respective spouse's who were at least 18 years old, were invited to participate. After the first contact with the proband, the first degree relatives were invited by phone to participate; all living relatives in the city of Baependi (urban and rural area) and surrounding cities were included in the study. To recruit the participants, the study was advertised through the provincial, religious, and municipal authorities, in local television, newspaper, and radio messages, through physicians, and by phone calls. For the physical examination, a clinic was established in an easily accessible sector of Baependi.

Information regarding family relationships, sociodemographic characteristics, medical history, and environmental risk factors such as physical activity, smoking habits, and alcohol use were evaluated through a questionnaire that was applied to each participant. The questionnaire was based on the WHO-MONICA epidemiological instrument, which was applied and filled out by research assistants specially trained for this task. Anthropometric measures such as weight and height were measured following the standardized procedures described earlier [8] and they were employed to define the body mass index (BMI) (kg/meters^2^).

The study protocol was approved by the ethics committee of the Hospital das Clínicas, University of São Paulo, Brazil, and each subject provided informed written consent before participation.

### Physical activity profiling

The physical activity profile of this population was delineated using four dimensions: sedentarism; weekly physical activity exclusively related to participation in sports activities (weekly PA); weekly physical activity among non-sedentary subjects only (weekly PA_NS); and the level of daily physical activity, including work activities (daily PA). Three global questions were used for data collection.

The first question was applied to assess the daily level of physical activity related to work and free-time activities, excluding sports. Four choices were presented, considering actions such as sitting, standing, walking, and object lifting in daily activities: (1) Do you have to remain seated to perform your activities? Do you walk while working?; (2) Do you walk a lot while performing your activities but do not need to lift or carry heavy objects?; (3) Do you walk and move many objects or go up and down stairs or an incline?; (4) Do your activities require major physical effort, for instance, do you lift or cut heavy objects? These choices represent levels of physical effort; extremely light (1), light (2), moderate (3), and vigorous (4). The subjects self-rated their daily level of physical activity, choosing the option that best represented their daily actions.

The second question was related to the practice of sports activity and its frequency: Do you take part in sports? In the case of an affirmative answer, how many days a week do you participate? Eight choices were presented: (1) I do not take part in sports; or I take part in sports (2) once a week; (3) twice a week; (4) three times a week; (5) four times a week; (6) five times a week; (7) six times a week; or (8) seven days a week. Subjects that chose the first option were classified as sedentary and the others, as active. This information was used to compose the sedentarism phenotype.

The last question registered the average time (in minutes) of each sport session for those subjects that participated in sports: What is the average duration of each sports session? A weekly PA phenotype was computed by multiplying the number of days a week in which the individual engaged in sports, assessed by the previous question, and the average time of each session.

Among the 1,857 individuals selected in the baseline phase of the Baependi Heart Study, 1,693 (98.9%) answered the questions that define the sedentarism and weekly PA phenotypes, and 1,675 (97.8%) answered the related question about daily physical activity. The weekly PA_NS data set is a subgroup of the weekly PA sample, which was composed of 515 non-sedentary subjects. Of the subjects that were classified as having the sedentarism phenotype because they did not practice sports, 283 subjects were engaged in work or free-time activities that required major physical effort (moderate or vigorous daily activities); they were therefore excluded from the data set to avoid misclassification. So, the final sample for the sedentarism analysis was composed of 1,410 subjects, 873 defined as sedentary and 537 defined as active.

### Statistical analysis

The sedentarism phenotype was analyzed as a dichotomous variable contrasting sedentary versus active subjects. The other traits were analyzed as continuous variables. Natural log-transformation was applied for weekly PA and weekly PA_NS traits to achieve the required normality assumption, followed by a new data assessment.

Familial correlations using the pairwise weighting scheme were computed using the FCOR program within the SAGE software package [9] for all main pair types of relatives available in the pedigrees.

Polygenic heritability estimates were calculated for all traits using the variance-components approach implemented in the SOLAR package [10]. In the most narrow sense, the heritability of a trait represents the proportion of the phenotypic variance attributable to additive genetic effects and is given by h^2 ^= σ^2^_a_/σ^2^_p_, where σ^2^_a _is the variance due to the additive effects of genes and σ^2^_p _is the phenotypic variance. For quantitative traits, the overall phenotypic variance was estimated from the observed distribution of trait values in the sample, and was partitioned into genetic and environmental components using the observed covariance among family members, as Ω = 2Φσ^2^_a _+ Iσ^2^_e_, where Ω is an nxn matrix of the n individuals in the data set; 2Φ is the structuring matrix of the coefficient of relationship; and I is an identity matrix that represents the structuring matrix for σ^2^_e_, the variance due to residual environmental factors. Covariates were not dealt with as a variance component, but rather as a modification to the trait mean, and covariate-specific trait means were used in the calculation of covariance among relatives. An extension of this basic model, the liability threshold model, was used to analyze discrete or categorical traits [11].

Household group analyses were also performed using the SOLAR system [10]. An additional variance parameter was added to model the effect of common environment, which is associated with any non-genetic factors shared between the individuals living in the same household at the time of study. So, the covariance matrix for pedigree, described above, was rewritten as Ω = 2Φσ^2^_a _+ Hσ^2^_c _+Iσ^2^_e_, where H is the structuring matrix for σ^2^_c_, the variance due to common environment effects; this matrix could contain zeros and ones depending upon whether an environment factor was shared or not between each pair of individuals. Using current residential addresses to define households, we obtained 740 nuclear families from the 95 families that are part of the Baependi Heart Study. Household effects were investigated in all polygenic models studied.

The SOLAR system handles categorical variables with more than two classes as quantitative traits. Thus, we applied a robust estimation approach implemented in SOLAR through the *tdist *procedure, to correct for bias of the heritability estimate of the daily PA phenotype.

Two models were fitted to the data: considering no covariate effects (model I); and considering age, sex, age^2^, and age by sex interaction effects, simultaneously (model II). Covariate screening determined the statistical significance of each effect; covariates with low significance levels (P ≥ 0.1) were removed from the final model, and the variance caused by all remaining covariates was computed. Household effects were included in all models.

Models with distinct genetic and environmental variance components were also employed to assess the evidence of heterogeneity among genders in the heritability estimates of the studied traits following the method described by Giolo et al. [12]. Assuming that the phenotypes in males and females are influenced by the same set of genes with distinct effects among genders, four situations regarding the genetic and environmental variance components were considered: homogeneity in both variance components, heterogeneity in at least one of the variance components, heterogeneity only in the environmental variance components, and heterogeneity only in the genetic variance components. Likelihood ratio tests were applied to define the models that presented the best fit to the data.

Models with distinct genetic and environmental variance components between the genders did not adequately fit to the daily PA dimension, even after a robust estimation approach implemented in SOLAR (*tdist *procedure) was employed to try to correct the bias of the heritability estimates. For this phenotype, the heritability estimates for the genders were obtained by considering males and females in separate analyses. Polygenic heritability estimates were calculated from model I (no covariate effects) and model II (with covariate effects) with the heritability estimation considering no heterogeneity among genders.

## Results

The physical activity profiles of the Baependi Heart Study families were assessed in four dimensions: sedentarism, weekly physical activity (weekly PA), weekly physical activity among non-sedentary subjects (weekly PA_NS) and the level of daily physical activity (daily PA) phenotypes. The anthropometric and sociodemographic characteristics of the sample are showed in Table 1. Among the 1,693 adults participating in the study, 43.4% were men and 56.6% women, ranging in age from 18 to 95 years. Because of the rural characteristic of the Baependi municipality, individuals remain active in fieldwork until an advanced age; so, the older subjects were maintained in our sample. The BMI was higher in females (25.3 kg/m^2^) than in males (23.4 kg/m^2^); so, on average, this population was within the healthy range (24.4 kg/m^2^). Overweight and obesity were observed in 26% of the female and 13% of the male subjects. The education level and socioeconomic status of the subjects were predominantly low; 58.3% went to school for ≤ 8 years and 84.7% belonged to classes D and E (low socioeconomic level). Table 2 illustrates the distribution of the sedentarism, weekly PA and daily PA phenotypes in the sample by gender and age groups. The weekly PA_NS phenotype subjects are a subgroup of the non-sedentary subjects in the weekly PA data set; therefore, this subgroup is not presented separately in Table 2. In our sample, the frequency of sedentary subjects (51.8% among males and 68.2% among females) is higher than non-sedentary subjects independent of gender, and shows a tendency to increase with age. The level of physical activity was predominantly low; in all age categories, most subjects exercised between 15 to 300 minutes a week; only 38 subjects aged from 18 to 24 years exercised more than 600 minutes a week. The routine of daily PA was predominantly light and independent of gender or age, excluding the 18-24 years age group, in which the frequencies of extremely light and light daily PA were similar (41.4%).

**Table 1:** Anthropometric and sociodemographic characteristics of the study population

	Men (n = 734)	Women (n = 959)	Total (n = 1,693)
**Age (years)**	44.6 (17.5)	43.9 (16.6)	44.2 (17.0)
**Weight (kg)**	69.3 (12.2)	63.3 (13.2)	65.9 (13.1)
**Height (cm)**	172.2 (7.3)	158.6 (7.2)	164.5 (9.9)
**BMI (kg/m^2^)**	23.4 (3.8)	25.3 (5.3)	24.4 (4.8)
**Education level (%)**			
unlettered	9.9	7.1	8.3
≤ 8 years (low)	59.0	57.9	58.3
9-11 years (moderate)	26.0	26.8	26.5
≥ 12 years (high)	4.8	8.2	6.7
**Socioeconomic status (%)**			
Class A + B (high)	0.9	0.6	0.8
Class C (moderate)	14.8	9.3	11.7
Class D + E (low)	81.7	87.0	84.7

BMI, body mass index; mean values (standard deviation) are shown for quantitative traits; the values expressed as percentages are for sociodemographic indicators.

**Table 2:** Distribution of phenotypes according to gender and age groups in the study population

	Sedentary	Non-sedentary	Weekly PA					Daily PA			
			
			0	15-300	301-600	601-900	900+	Extremely light	Light	Moderate	Vigorous
	
	n (%)	n (%)	n (%)	n (%)	n (%)	n (%)	n (%)	n (%)	n (%)	n (%)	n (%)
**Gender**											
Male	279 (51.8)	260 (48.2)	474 (65.3)	178 (24.5)	55 (7.6)	16 (2.2)	3 (0.4)	200 (27.5)	246 (33.8)	108 (14.9)	173 (23.8)
Female	594 (68.2)	277 (31.8)	682 (72.2)	199 (21.0)	45 (4.8)	15 (1.6)	4 (0.4)	291 (30.7)	541 (57.1)	98 (10.3)	18 (1.9)

**Age (years)**											
18-24	117 (52.5)	106 (47.5)	143 (57.9)	69 (27.9)	19 (7.7)	12 (4.9)	4 (1.6)	101 (41.4)	101 (41.4)	16 (6.6)	26 (10.6)
25-39	237 (60.8)	153 (39.2)	306 (67.6)	116 (25.6)	26 (5.7)	4 (0.9)	1 (0.2)	134 (29.4)	214 (46.9)	51 (11.2)	57 (12.5)
40-54	260 (65.0)	140 (35.0)	382 (74.2)	93 (18.1)	29 (5.6)	9 (1.7)	2 (0.4)	122 (23.5)	243 (46.8)	78 (15.1)	76 (14.6)
55+	259 (65.2)	138 (34.8)	325 (71.3)	99 (21.7)	26 (5.7)	6 (1.3)	0 (0.0)	134 (29.4)	229 (50.2)	61 (13.4)	32 (7.0)

Weekly PA, weekly physical activity (minutes); daily PA, level of daily physical activity.

Familial correlations estimated in the various types of relatives are presented in Table 3. Significant non-zero correlations were observed among siblings for the weekly PA, weekly PA_NS, and daily PA phenotypes (0.08-0.32), and in the avuncular relationship for weekly PA_NS trait (0.15). These results indicate that subjects of the same generation who are genetically and closely related tend to be more similar in their physical activity habits than subjects of different generations. There was no familial correlation for the sedentarism phenotype.

**Table 3:** Interclass and intraclass correlations in different pairs of relatives for the different phenotypes

Relationship type	Sedentarism	Weekly PA	Weekly PA_NS	Daily PA
	
	Correlation (s.e.)	Correlation (s.e.)	Correlation (s.e.)	Correlation (s.e.)
parent:offspring	0.05 (0.03)	0.00 (0.03)	0.06 (0.09)	0.02 (0.03)
sibling	0.03 (0.03)	0.14 (0.03)***	0.32 (0.08)***	0.08 (0.03)**
grandparent	0.06 (0.06)	0.04 (0.06)	-0.04 (0.17)	0.06 (0.05)
avuncular	0.02 (0.02)	0.05 (0.03)	0.15 (0.07)*	0.02 (0.02)
cousin	0.05 (0.03)	0.05 (0.03)	0.06 (0.08)	0.01 (0.03)
mother:father	0.07 (0.07)	0.05 (0.06)	0.16 (0.19)	0.08 (0.06)

s.e., standard error; * p ≤ 0.05; ** p ≤ 0.01; *** p ≤ 0.001.

Two polygenic models, model I (no covariate effects) and model II (with covariate effects) were fitted to the data in all the analyses. Only model I fitted the weekly PA_NS data. Model II was also applied to the weekly PA_NS data, but the covariates presented no significant effect on this phenotype. We did not observe significant household effects for any of the studied traits, irrespective of the model used for the analysis.

Heritability estimates were intermediate (0.35) for weekly PA_NS and low for sedentarism, weekly PA, and daily PA (0.09 to 0.14 for model I, and 0.10 to 0.12 for model II). All covariates (age, sex, age^2^, and age by sex interaction effects) showed significant effects on the sedentarism trait, and their inclusion in the model slightly decreased the heritability estimate, suggesting that a part of the polygenic effect might be explained by the covariates that were used. Similar results were produced by the covariates sex, age^2^, and age by sex interaction for the daily PA phenotype. For weekly PA, only the sex covariate had no significant effect; the inclusion of the other covariates into the model slightly increased the value of the heritability estimate. Although the adjustment of the model for the covariates was significant, especially for the weekly PA phenotype, the covariates contributed to only a small proportion (2.9-4.4%) of the total phenotypic variance of each trait (Table 4).

**Table 4:** Parameter estimates for the sedentarism, weekly PA, and weekly PA_NS phenotypes in the Brazilian families

Phenotype	Model	No heterogeneity			Heterogeneity in variance components by gender						Selected model Variance with heterogeneity
			
		h^2 ^(s.e.), p value	σ^2^_g_	σ^2^_e_	h^2^_m_	h^2^_f_	σ^2^_g, m_	σ^2^_e, m_	σ^2^_g, f_	σ^2^_e, f_	
**sedentarism**	No covariate	0.14 (0.07), p = 0.0104	16.45	99.99	0.05	0.22	5.16	99.98	0.27	0.95	Genetic and environmental
	Age, sex, age × sex, age^2^, age^2 ^× sex	0.12 (0.07), p = 0.0218	1.22	8.48	0.12	0.12	1.22	8.48	1.22	8.48	No heterogeneity

**weekly PA**	No covariate	0.09 (0.04), p = 0.0019	0.55	5.44	0.11	0.09	0.59	5.90	0.59	5.02	Environmental
	Age, age × sex, age^2^, age^2 ^× sex	0.11 (0.04), p = 0.0004	0.62	5.20	0.11	0.11	0.62	5.20	0.62	5.20	No heterogeneity

**weekly PA_NS**	No covariate	0.35 (0.11), p < 0.0001	0.24	0.43	0.35	0.35	0.24	0.43	0.24	0.43	No heterogeneity

Weekly PA, weekly physical activity; weekly PA_NS, weekly physical activity among non-sedentary subjects; h^2 ^(s.e.), heritability estimate (standard error); σ^2^_g_, polygenic variance estimate; σ^2^_e_, environmental variance estimate; h^2^_m_, heritability estimate for males; h^2^_f_, heritability estimate for females; σ^2^_g,m_, polygenic variance estimate for males; σ^2^_e,m_, environmental variance estimate for males; σ^2^_g,f_, polygenic variance estimate for females; σ^2^_e,f_, environmental variance estimate for females.

There was no heterogeneity in the variance components by gender for the sedentarism, weekly PA, weekly PA_NS phenotypes when the models were adjusted by covariates. Using the model with no covariate effects, heterogeneity by gender was observed for the sedentarism trait in both (genetic and environmental) variance components, with heritability higher in females (0.22) than in males (0.05). For the weekly PA trait, heterogeneity by gender was observed in only the environmental variance component (heritability estimates were 0.09 in females and 0.11 in males). Because the models with distinct genetic and environmental variance components among the genders did not fit adequately to the daily PA phenotype, the heritability estimates were obtained from data sets that were separated for males and females, considering models I (no covariate effects) and II (with covariate effects). In males (727 subjects), the heritability estimates were 0.22 ± 0.07 and 0.21 ± 0.07 for models I and II respectively. The age^2 ^covariate represented about 2% of the phenotypic variance of this trait, and its inclusion into the model slightly decreased the heritability estimate, following the trend observed in the analysis with no heterogeneity among genders. In females (947 subjects), there were no significant covariate effects for the daily PA trait and the heritability estimate was 0.11 ± 0.06. For model I, the heritability estimate obtained for males (22%) was exactly twice that for females (11%). There were no significant household effects for the daily PA phenotype in these analyses.

The results of heritability analyses performed with or without heterogeneity in the variance components are shown in Tables 4 and 5.

**Table 5:** Heritability estimates for the daily PA phenotype from analysis with separate data sets by gender

Gender	No heterogeneity		Heterogeneity by gender	
	
	Model	h^2 ^(s.e.), p value	Model	h^2 ^(s.e.), p value
Male	No covariate	0.12 (0.04), p = 0.0001	No covariate	0.22 (0.07), p = 0.0002
	Sex, age × sex, age^2^	0.10 (0.04), p = 0.0005	Age^2^	0.21 (0.07), p = 0.0003

Female	No covariate	0.12 (0.04), p = 0.0001	No covariate	0.11 (0.06), p = 0.0204
	Sex, age × sex, age^2^	0.10 (0.04), p = 0.0005		

h^2 ^(s.e.): heritability estimate (standard error).

## Discussion

In this study, the physical activity profile of the sample of Brazilian adults in the Baependi Heart Study was investigated using data collected by questionnaires based on the WHO-MONICA epidemiological instrument. Baependi is located in a rural area of Brazil and its economy is based on agriculture and this, for the most part, explains the low sociodemographic indicators observed in the sample and described in Table 1. Although the obesity prevalence was similar to the 2006 rate observed in the Brazilian population as a whole [13], the overweight prevalence in the sample was lower (26% in the study population versus 34.1-48.3% in the Brazilian population). Obesity is a complex disease determined by genetic predisposition and environment factors such as diet and sedentary behavior. Considering the low level of weekly physical activity observed in this sample, the lower overweight rate is probably associated with the level of daily activities and a healthy diet that is commonly observed among the inhabitants of rural areas. Subjects predominantly classified their daily activities as light, but the concept of light work in rural and urban areas is very different. Field work demands high physical effort and this probably explains the low overweight prevalence that was observed. In general, the level of weekly physical activity was higher in males than females and this declines with increasing age, consistent with previous studies [14] (Table 2).

Significant intraclass and interclass correlations were observed for sibling and avuncular relationships. This result indicated that environmental factors and lifestyles shared by persons of the same generation who are genetically and closely related play a significant role in the phenotypic variance of physical activity. Significant correlations among siblings for physical activity phenotypes have also been found in other populations, although the results are not directly comparable because of divergence in the studied phenotypes and in sample characteristics [15,16]. One of these studies did assess the avuncular interclass correlation, but it was not found to be significant in any of the studied phenotypes [15]. We have yet to explain the shared environment effects observed for the avuncular correlation in the weekly PA_NS phenotype. In our study, no significant parent-offspring correlation was found, although similarity in their physical activity habits was commonly detected. This result indicated that either different genetic factors were modulating physical behavior in the two generations, or that there were generation-specific environmental factors promoting more resemblance within generations than across generations [17]. Another possible explanation is that of a drastic change in the rural Brazilian society occurred during the last decades and this may have diluted the effects.

Several approaches have been used to model the genetic contributions to physical activity phenotypes. Twin [18,19] and family [20,21] studies have been employed to dimension genetic factors, associations [7,22,23], linkages [23-25], and animal [26,27] studies have contributed to the identification of candidate genes and metabolic alterations involved in the determination of physical activity phenotypes. Animal studies have also been used to describe the associations between physical activity phenotypes and other risk factors for cardiovascular diseases, such as obesity and type 2 diabetes. Different genomic regions have shown linkages with distinct activity phenotypes, suggesting that each of the phenotypes might be influenced by specific mechanistic pathways. In human, significant linkage signals have been identified on chromosomes 2p22-p16, 7p11.2, 18q, and 20q13.1 for physical inactivity, on 13q22-q31 and 18q for total physical activity, on 18 q for light and moderate activity, on 4q28.2, 7p11.2, 9q31.1 and 13q22-q31 for moderate and strenuous physical activity, and on 11p15 and 15q13.3 for time spent in physical activity [24,25]. The 18q region, which has been linked to several physical activity phenotypes, flanks a candidate gene, *MC4R*, whose mutations have been associated with the development of obesity [28]. *MC4R *is an important component of the regulation of energy balance, which is maintained by controlling energy intake, as food, and energy expenditure, as physical activity and metabolism. Association and animal studies with knockout and transgenic mice have confirmed the connection between *MC4R *and activity phenotypes and its involvement in the regulation of spontaneous and stimulus-induced activity [22,27]. Other genes, such as gastrin-releasing peptide (*GRP*), striatin, transcripts encoding the endothelin B-receptor, and the cocaine- and amphetamine-regulated transcript (*CART*) have also been suggested as potential candidates to explain the variation of physical activity phenotypes [22,24,25].

The sedentarism, weekly PA, weekly PA_NS, and daily PA phenotypes were significantly influenced by additive genetic effects in the Baependi Heart Study population. The heritability estimates for physical activity (0.09-0.35) were lower than those found in twin studies (0.35 to 0.83) [18,19,29,30], but they were within the range of estimates derived from family studies (0.06 to 0.66) [14-16,20,21,25,31]. The values for the heritability estimates in our study were lower for the weekly PA trait (0.19 and 0.26), and higher (0.23-0.60) for the sedentarism and daily PA traits than those reported in the literature [15,16,20,21,25,32,33]. In these studies, the experimental design varies in the methodology for parameter estimation, sample characteristics, and, mainly, in phenotype definition; therefore, a direct comparison of the heritability estimates is very difficult. In addition, only two of the studies were conducted with Mexican-American families [14,20] that would have genetic backgrounds most similar to the high admixed Brazilian population; nevertheless, the phenotype acquisition and definition are distinct in relation to our study.

Genetic effects can be confounded by within-family transmission of behavioral patterns, thus household effects in the families of this population were investigated in all tested models. None of the analyzed phenotypes were significantly affected by a common household environment, consistent with a previous study [5]; however, the significance of the household effect on physical activity dimensions is inconsistent across previous studies. In the literature, shared environment components have been shown to have significant effects for both genders [29], in only one gender [30], in only one age group [34], or in specific phenotypes in a population [15]. These inconsistencies are not surprising, as potential household effects may be heterogeneous and may represent unmeasured factors, such as diet, attitudes towards physical activity, family composition, neighborhood characteristics and cultural background. In addition, current residential addresses were used in our study to identify households and this could not capture long-term cumulative household effects, particularly those related to the formative years of the subjects [21].

Some studies have suggested that distinct genetic factors can influence the phenotypic expression of physical activity traits in males and females [29,35], but this is still not well established. No evidence for a different set of physical activity genes in the two genders has been described in the literature [30,36]. The heterogeneity in the results of the various studies indicates that knowledge about the genetic and environmental components of the physical activity variation is still incomplete. To assess this problem, we employed models in which heterogeneity in males and females was allowed in both variance components, in only the environmental variance component, or in only the genetic variance component. Significant gender differences were detected only with the models with no covariate effects. In the sedentarism phenotype, we observed heterogeneity in both variance components, with heritability estimates higher in females (0.22) than in males (0.05), and in the weekly PA trait, heterogeneity was seen in only the environmental variance component (heritability of 0.09 in females and 0.11 in males). There was no heterogeneity in variance components by gender for the weekly PA_NS trait. The models did not fit adequately for the daily PA trait, even when a robust estimation approach was applied, probably because the SOLAR package handled it as a quantitative trait. The heritability estimation was also performed for female and male data sets separately. Only the model with no covariate effects fitted to the female data. Using this model, the estimate in males was twice that for females. Considering the total phenotypic variance observed in males and females (1.241 and 0.435, respectively), we found that the level of daily physical activity in both genders was predominantly influenced by the environmental variance component, and this was higher in males than females. Similar [29,35] and discordant [30,36] results have been reported in the literature, suggesting that the heterogeneity in the variance components by gender is still not well defined for physical activity phenotypes.

Our study had some limitations. The first limitation was related to the questionnaire applied to assess the physical activity profile of this population. This questionnaire was based on the WHO-MONICA epidemiological instrument, but this has not yet been validated. Although our results are consistent with previous studies, they must be used with caution. Further, our study was based on self-reported answers to the questions and may suffer from phenotypic misclassifications in the data that could lead to underestimated heritability or to results with large standard errors; however, this is a common feature of other work on this topic. This issue could explain the low heritability estimates obtained for the sedentarism and daily PA phenotypes in our study. More objective devices such as accelerometers or pedometers have been proposed to collect physical activity data, but their application in large samples is difficult. The second limitation was the sample size. The data set used in this study is small in terms of subjects evaluated and controlled variables. The Baependi Heart Study is a longitudinal study and the first follow-up, which will permit a more effectively assessment of the physical activity profile of this population, has been started.

## Conclusions

In conclusion, employing variance-components approaches, we have estimated the genetic influences on physical activity phenotypes in the Brazilian families that were part of the Baependi Heart Study. Heritability estimates for the sedentarism, weekly physical activity (weekly PA), weekly physical activity among non-sedentary subjects (weekly PA_NS), and level of daily physical activity (daily PA) traits were significant and from low to intermediate magnitude. Significant evidence for heterogeneity in variance components by gender was observed for the sedentarism and weekly PA phenotypes, but not for the weekly PA_NS trait. In the study population, the daily PA trait was predominantly influenced by environmental factors that had higher effects in males than in females. These data will add to the knowledge of the physical activity phenotypes in the Brazilian population and are concordant with the notion of significant biological determination in active behavior.

## Competing interests

The authors declare that they have no competing interests.

## Authors' contributions

ARVRH performed the data analysis and interpretation, and wrote the manuscript. SRG, JPS, and MA participated of the concept and design of the study, and provided statistical support for the data analysis and interpretation. CMO and ROA performed the data acquisition and analysis. JEK participated in the concept and design of the study and revised the manuscript. ACP participated in the concept and design of the study, provided support for data analysis and interpretation, and revised the manuscript. All authors read and approved the final manuscript.

## Pre-publication history

The pre-publication history for this paper can be accessed here:

http://www.biomedcentral.com/1471-2350/12/155/prepub
